# Surface Engineering and Patterning Using Parylene for Biological Applications

**DOI:** 10.3390/ma3031803

**Published:** 2010-03-15

**Authors:** Christine P. Tan, Harold G. Craighead

**Affiliations:** 1 Department of Biomedical Engineering, Cornell University, Ithaca, New York, NY 14853, USA; E-Mail: cpt7@cornell.edu; 2 School of Applied and Engineering Physics, Cornell University, Ithaca, New York, NY 14853, USA

**Keywords:** parylene, micropatterning, surface modification, [2.2]paracyclophane, biomaterial, bioactive, biomolecular, nanobiotechnology, microarray, microfluidic

## Abstract

Parylene is a family of chemically vapour deposited polymer with material properties that are attractive for biomedicine and nanobiotechnology. Chemically inert parylene “peel-off” stencils have been demonstrated for micropatterning biomolecular arrays with high uniformity, precise spatial control down to nanoscale resolution. Such micropatterned surfaces are beneficial in engineering biosensors and biological microenvironments. A variety of substituted precursors enables direct coating of functionalised parylenes onto biomedical implants and microfluidics, providing a convenient method for designing biocompatible and bioactive surfaces. This article will review the emerging role and applications of parylene as a biomaterial for surface chemical modification and provide a future outlook.

## 1. Introduction

The ability to manipulate biological interfaces and systems is useful in biomedical applications. Surface chemical modification is often necessary to mimic biologically relevant environments. In complex organisms, the signaling between biomolecules in the extracelullar matrix occurs on nanoscale dimensions, while cell-cell interactions and tissue architecture are controlled at the microscale level. Micropatterning is a tool for surface biochemical modification and involves the spatial manipulation and deposition of biomolecules with micro- and nanometre length-scale precision. Recent developments in micropatterning [1,2,3,4,5,6,7] have propelled the engineering of biological microenvironments for tissue engineering, micro total analysis systems (biosensors, microfluidics and microarrays), and fundamental biophysical studies [8,9,10,11,12]. Moreover, surface chemical modification via surface coatings is a method to create biocompatible or biologically active surfaces for drug delivery systems, biomedical implants and prostheses [13,14,15]. Ideally, the surface coating can be applied that contains chemical functionalities suitable for immobilising proteins, drugs or anti-coagulant agents. With these growing demands, there is a challenge to discover and develop new biomaterials for surface engineering in biology.

Parylene is a relatively new biomaterial for surface engineering, although it has been mostly used for several decades in non-biological applications. This review will address the role of parylene in surface modification for biological applications in five parts. First, a background on parylene, its deposition process and material properties will be introduced. Second, the utility of parylene as a micropatterning stencil template will be discussed. This is followed by a review of the applications of parylene reactive coatings for surface modification. Next, parylene as a biologically compatible material for creating surface microstructures, such as tubes, microfluidics, membranes and thin films will be discussed. Finally, a future outlook will be provided on the opportunities and potential of parylene as a biomaterial of choice for surface engineering in the field of biomedicine and nanobiotechnology.

## 2. Background

### 2.1. History and Chemical Vapour Deposition of Parylene

**Figure 1:**
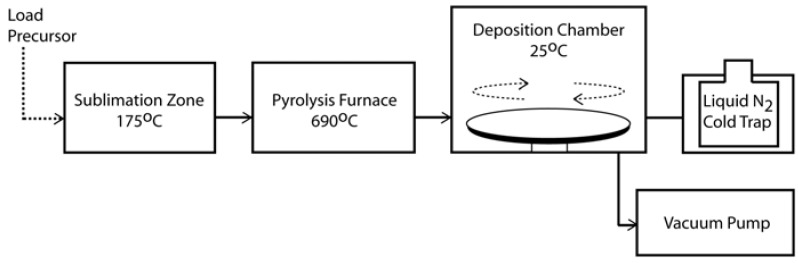
CVD polymerisation of parylene-C. The precursor is loaded into the sublimation zone (175 °C). The sublimed form of the precursor is transported into the furnace (690 °C) for pyrolysis into the reactive monomer, which finally polymerises into parylene spontaneously upon cooling in the deposition chamber (25 °C). The substrate stage is usually rotated for increasing the uniformity of deposition.

**Figure 2:**
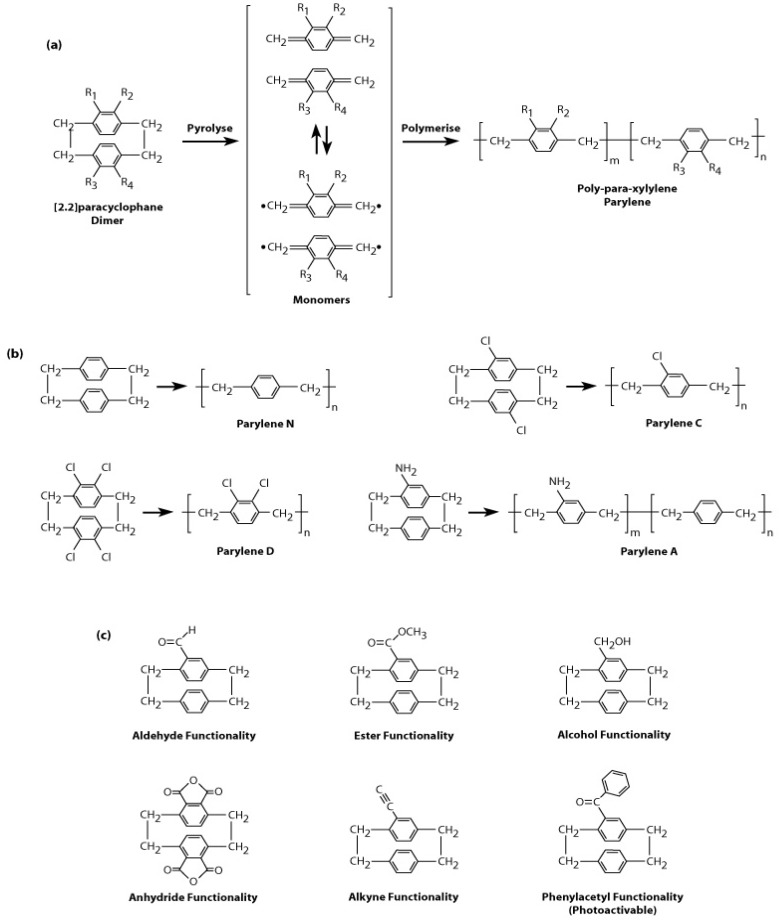
(a) Precursor [2.2]paracyclophane and its polymerisation into parylene. (b) Types of commercially available precursors and their parylenes. (c) Substituted forms precursors synthesised by research laboratories.

Parylene (or poly-para-xylylene) was initially observed as the product of vacuum thermal decomposition (pyrolysis) of the common solvent, para-xylene [16]. Despite the relatively high temperatures 700–900 °C, the yield of parylene film was low. Today, parylene can be deposited more efficiently and easily via a chemical vapour deposition (CVD) process developed by Gorham [17]. In this CVD process, the dimer precursor [2.2]paracyclophane (or di-para-xylylene) is first vapourised and then undergoes vacuum pyrolysis at temperatures above 550 °C to yield the reactive monomer. The monomer adsorbs to the substrate surface and spontaneously polymerises at room temperature to form linear, high molecular weight parylene films. Figure 1 shows the typical CVD process parameters for depositing parylene-C using commercial systems, while Figure 2a shows the chemical reactions underlying the CVD process and the chemical structures of [2.2]para-cyclophane and parylene. The Gorham process enables control of the deposition parameters and full conversion of the precursor into parylene. It is appealing that the substrates are kept at room temperature during deposition, enabling parylene to be coated onto a variety of heat-sensitive substrates without thermal damage.

The kinetics of the parylene CVD process have been elegantly reviewed by Fortin and Lu [18]. A key feature is that parylene deposition is under kinetic control. The rate-limiting steps of kinetically controlled CVD are: (1) the adsorption of the monomer onto the substrate, (2) surface migration and possibly bulk diffusion of the monomer, and (3) the chemical reaction (initiation or propagation) at the surface. Selective deposition of parylene onto localised regions of the substrate can be realised by intervening in any of these rate-limiting steps. For example, several research groups have exploited this to form patterned films of parylene by using either transition metals and their salts to deactivate the monomer reaching the substrate surface [19,20,21], or localised heating to vary deposition rates at the surface [22,23,24,25]. These methods are alternatives to lithography for patterning parylene films. Due to the low sticking coefficient (<1 × 10^-3^) at room temperature [18], parylene is known for its excellent conformal coating of the substrate, even trenches, which is highly attractive for coating biomedical stents. However by suitably engineering the aspect ratios of the trenches and controlling parylene thickness, the otherwise conformal parylene film can be pinched off to form microfluidic tubes [26,27].

### 2.2. Types of Parylene

The general chemical structure of [2.2]para-cyclophane is shown in Figure 2a. A variety of substituted [2.2]para-cyclophanes exist, whereby functional groups may be introduced into any of the R_1_, R_2_, R_3_ and R_4_ positions. These functional groups are not modified during the CVD process and allow for the deposition of functionalised parylene films with tailored chemical, mechanical, electrical and optical properties. Functionalised parylene coating is a versatile and simple method to introduce chemical functionalities to a surface, for example a biomedical implant, without the use of external organic solvents and catalysts, thereby reducing contamination and improving biocompatibility. Various [2.2]para-cyclophanes that are commercially available and their parylenes are shown in Figure 2b, and substituted [2.2]para-cyclophanes that have been synthesised by research laboratories [15,28] are shown in Figure 2c. Improvements in the chemical synthesis of the substituted precursor have led to parylene films with active functional groups such as carbonyl [29], amine [30], aldehyde [31], ester [32], anhydride [32], alkyne [33], alcohol [34], and photoactivable phenylacetyl [35,36]. These active groups can further undergo chemical reaction to allow covalent immobilisation of drugs [37], disaccharides [31], proteins [38] and polyethylene oxide [36] onto the parylene coated surfaces; these specific applications will be discussed later in the section on reactive parylene coatings. For example, alkynes are of great interest for chemical coupling with azides via “click” chemistry, which has been used in drug discovery, nanobiotechnology and surface chemistry [39]. Furthermore by mixing the ratios of two different types of substituted precursor, a “multipotent” film of parylene containing two different functional groups may also be deposited [40].

### 2.3. Material Properties and Applications of Parylene

Some material properties of commercial parylene are highlighted in Table 1. Parylene coatings have been used to protect electronic printed circuit boards (PCB) boards, because parylene possesses low permeability to water and gases and forms highly conformal pinhole-free coatings. Parylene is amenable to micromachining by photolithography processes, and has been used as dielectric layers in semiconductor industries, as well as anti-stiction coatings for microelectromechanical systems (MEMS) due to its low coefficient of friction [41]. Unique properties such as biocompatibility, the pin-hole free and highly conformal nature of the parylene films, and low water absorption (swelling), have led to their recent adoption for biomedical applications, such as stents, neural electrodes, cardiac pacemakers, intra-oral magnets [42], and high-fidelity micropatterning of biomolecules [43].

**Table 1:** Some material properties of commercial available parylenes, data collected from reference [44], data specification sheets from Specialty Coating Systems and Uniglobe Kisco.

	Parylene-N	Parylene-C	Parylene-D
Density (g/cm^3^)	1.11	1.289	1.418
Refractive Index	1.661	1.639	1.669
Melting Point (°C)	420	290	380
Dielectric Constant (60Hz)	2.65	3.15	2.84
Tensile Modulus (GPa)	2.4	3.2	2.84
Elongation to Break (%)	30	200	10
Static Coefficient of Friction	0.25	0.29	0.31
Dynamic Coefficient of Friction	0.25	0.29	0.31
Water Absorption (% in 24h)	<0.1	<0.1	<0.1
Oxygen Gas Permeability (cc.mm)/(m^2^.day)	15.4	2.8	12.6
Water Vapour Transmission Rate (g.mm)/(m^2^.day)	0.59	0.08	0.09
Water Contact Angle	79°	87°	97°

### 2.4. Post-Coating Surface Modification of Parylene

**Figure 3:**
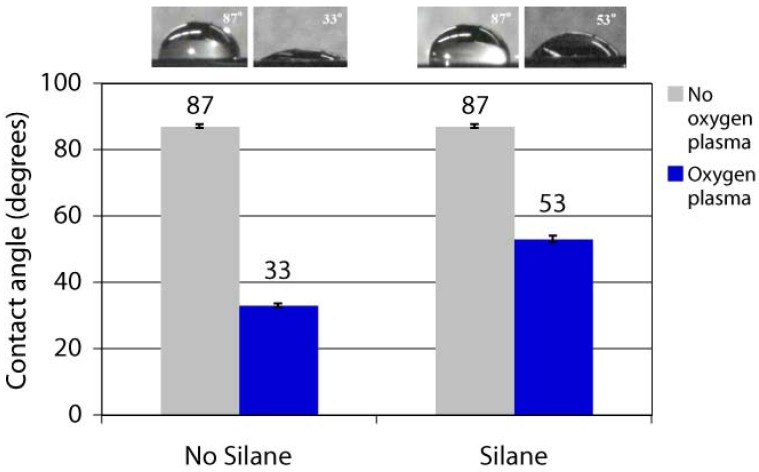
Water contact angles of parylene following different treatments: with oxygen plasma etch and/or silanisation with a hydrophilic aminopropyltrimethoxysilane. Reproduced with permission from reference [47]; proceedings published by the Chemical and Biological Microsystems Society, 2008.

The surface free energy of untreated parylene-C films is 19.6 mN/m, corresponding to a hydrophobic surface with a high water contact angle. Different functionalised parylenes have different surface properties (hydrophobicity or hydrophilicity), as seen by the different water contact angles in Table 1. This opens up opportunities for controlling and tailoring surface hydrophilicity through the use of parylene coatings. Parylene-C films exposed to oxygen plasma can become permanently hydrophilic due to the destruction of chemical bonds at the parylene surface, and the surface free energy can increase to more than 60 mN/m (water contact angle decrease to lower than 45°) after just 20 seconds of oxygen plasma [45]. This method of modifying the hydrophilicity of parylene has been exploited for improving cell adhesion to parylene-C surfaces [46]. It has been observed that untreated parylene films are resistant to silanisation, while oxygen plasma treated parylene surfaces can be silanised using via vapour deposition of aminopropyltrimethoxysilane, as shown by the water contact angles in Figure 3 [47]. A similar mechanism has been demonstrated for surface acoustic wave (SAW) devices, whereby the SAW surface was first coated with parylene-C, then oxygen plasma treated and silanised with (3-glycidyloxypropyl)trimethoxysilane for covalently immobilising aminodextran and folic acid as biosensors [48].

## 3. Micropatterning Using Parylene “Peel-Off” Stencils

**Figure 4:**
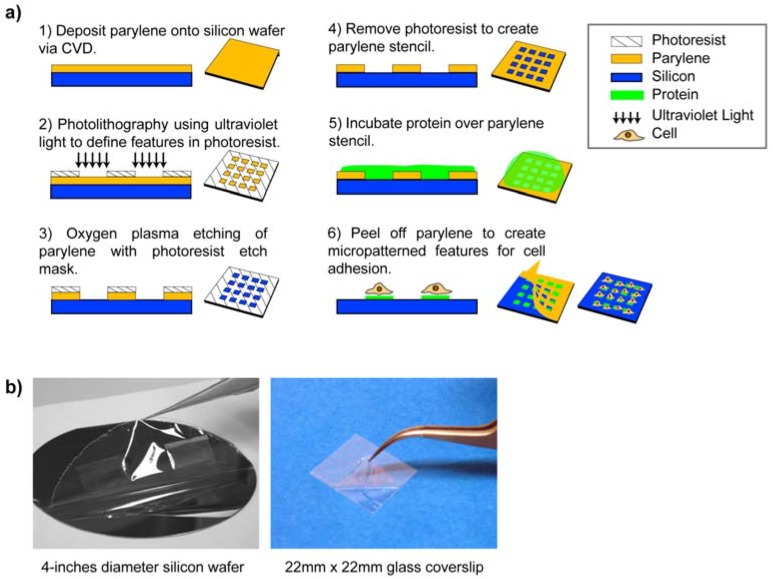
(a) Fabrication process of parylene-C stencils and the parylene “peel-off” micropatterning process. (b) Parylene “peel-off” has been used to micropattern large areas such as 4-inches diameter silicon wafer and 22 mm × 22 mm glass coverslips. Figures adapted with permission from reference [7], copyright 2010 American Chemical Society; and from reference [53] (DOI:10.1039/b908036h), reproduced by permission of The Royal Society of Chemistry.

Surface micropatterning using parylene-C “peel-off” stencils was first pioneered by Ilic and Craighead [43] for patterning proteins and cells on a surface. These stencils were microfabricated using standard photolithography processes illustrated in Figure 4a (steps 1–4). Briefly, parylene was first coated onto a silicon wafer and a photoresist layer was spun on top of the parylene. Next, the photoresist was exposed by ultraviolet light and developed, creating features (openings) in the photoresist. The photoresist layer then served as an etch mask during oxygen plasma etching of the underlying parylene layer in the openings. Because photoresist etches at a similar rate as parylene, the photoresist layer has to be thicker than the parylene layer. Finally, the excess photoresist was removed by acetone and isopropanol to leave the parylene stencil on top of the wafer. Steps 5–6 of Figure 4a outline the procedure for micropatterning. The biomolecule of interest (in this case protein) was incubated onto the parylene stencil coated wafer surface. Thereafter, the parylene stencil can be mechanically “peeled off” to spatially pattern proteins in the localised regions defined by the openings. Thus, the parylene stencil also served as a protective coating for excluding the regions outside the openings. In this initial demonstration, arrays of poly-l-lysine, antibodies, mammalian mast cells and bacterial *E.coli* cells were micropatterned. The parylene “peel-off” approach can be employed in different configurations for micropatterning. For example, parylene “peel-off” can also be used for patterning cell co-cultures by sequentially patterning the first cell type, then peeling off the parylene and incubating with a second cell type [49]. Multilayer parylene stencils [50,51] and ultra-thick re-usable parylene stencils [52] have been also developed.

There are two major advantages of parylene stencils. First, parylene is pinhole-free, chemically inert and resists swelling in aqueous solutions. These characteristics allow for biomolecules to be patterned with high fidelity and uniformity using the stencil. Microcontact printing (mCP) is a popular micropatterning technique utilising elastomeric polydimethylsiloxane (PDMS) stamps that can deform unevenly under pressure, shrink during curing, or swell in solutions. Parylene “peel-off” alleviates the problems associated with mCP, and increases the uniformity of micropatterning. Second, the parylene “peel-off” micropatterning approach does not require harsh chemicals and can be performed completely in aqueous environments, which preserves the conformation and activity of sensitive biological species. Other advantages of using parylene stencils for micropatterning include the ability to pattern on a variety of substrate surfaces with large areas rapidly. Figure 4b shows the parylene “peel-off” method used in micropatterning 4-inches diameter silicon wafer and 22 mm × 22 mm glass coverslips.

### 3.1. Multi-Component Protein Arrays with Nanoscale Resolution

**Figure 5:**
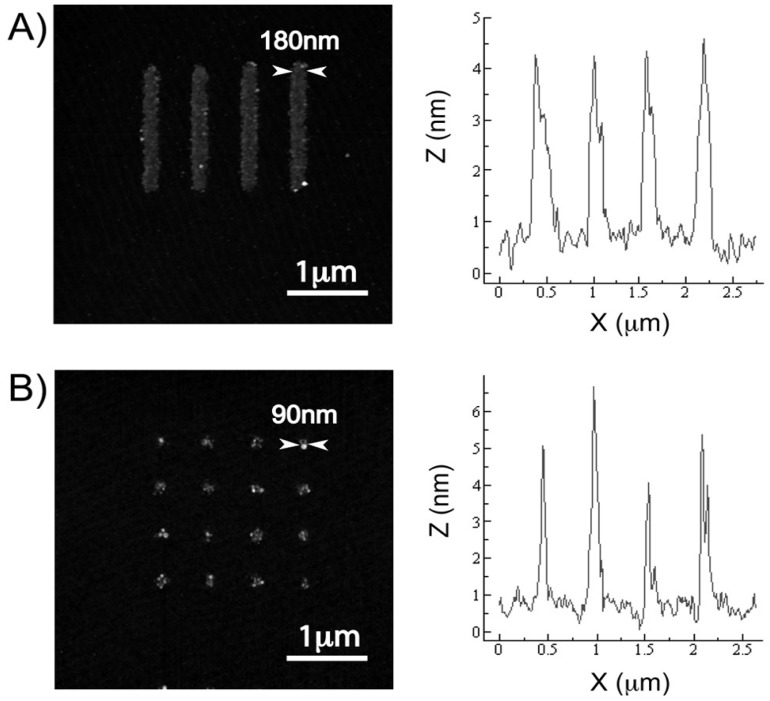
Atomic force microscopy images and the cross-sectional profiles of patterned fibronectin nanoarrays—(a) 180nm lines and (b) 90nm spots. The fibronectin patterns were replicated with high fidelity from the parylene templates. The cross-sectional profiles were taken from a span across four array features on each image, and the full width at half maximum of each peak was measured as the feature width. The heights of the patterned fibronectin features were relatively uniform at 4–5nm. Reproduced with permission from reference [7]. Copyright 2010 American Chemical Society.

Earlier work with the parylene “peel-off” stencil approach typically show the micropatterning biomolecules and cells with micrometre feature sizes. The micropatterning process was in part limited by the use of the thick resist etch masks that were etched at the same rate by oxygen plasma as parylene. Recently, Tan and co-workers have reported a nanofabrication process that uses an ultra-thin aluminum layer as the etch mask, thereby enabling parylene stencils with sub-100nm openings to be fabricated [7]. Highly uniform nanoscale features (arrays of lines and spots) of fibronectin were patterned as shown in Figure 5, demonstrating for the first time that parylene “peel-off” could be used for patterning biomolecules with nanoscale resolution.

**Figure 6:**
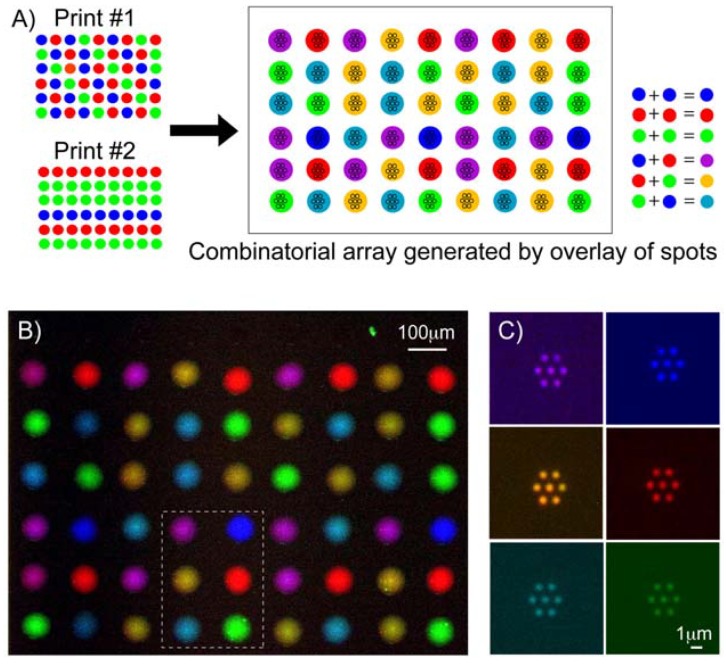
The PNP process can generate combinatorial biomolecular nanoarrays. (a) Schematic diagram showing the process of superimposing a second inkjet print-run immediately over the first print to generate six different combinations of antibodies from an initial pool of three separate antibodies. (b) Pseudo-colour merged fluorescence image showing the combinatorial array that can be inkjet printed onto the parylene template. (c) Antibodies nanoarrays of six different biomolecular combinations were generated after parylene peel-off, corresponding to the spots demarcated within the boxed region in (b). Reproduced with permission from reference [7]. Copyright 2010 American Chemical Society.

Additionally, this work combines inkjet printing with parylene “peel-off” (Print-and-Peel [PNP]) [7]. PNP involves the alignment of an inkjet printed spot with a set of openings in the parylene. After peeling off the parylene, protein arrays with uniform nanoscale feature sizes and shapes are obtained. This could be useful for cleaning up imperfect inkjet printed spots, as well as extending the resolution of inkjet printing to nanoscale dimensions. Through the use of inkjet printing, PNP enhances the utility of the parylene “peel-off” approach to pattern multi-component proteins (potentially hundreds to thousands) and their combinations on a single chip as shown in Figure 6. This had not been previously achieved, since typically one type of biomolecule is bath-incubated on the parylene stencil surface at a time. This approach is potentially a convenient alternative to achieve rapid multiplexing of patterning biomolecular nanoarrays over large patterned areas, compared to dip-pen lithography that utilises a relatively more complex and slower atomic force microscope system to write nanoscale features. The ability to pattern multi-component biomolecular arrays with nanoscale resolution will open new possibilities for precise placement of biomolecules with nanometre length-scale control in tissue engineering and in high-resolution biophysical imaging studies.

### 3.2. Controlling Deoxyribonucleic Acid (DNA) Microarray Spot Morphology

**Figure 7:**
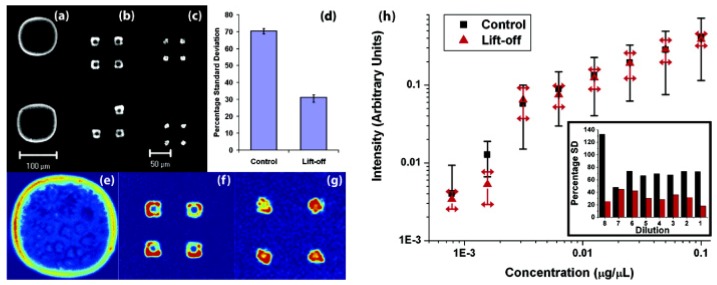
Confocal imaging showing the improvement of spot uniformity after hybridisation obtained with polymer liftoff arrays. (a-c) Raw images from control and polymer liftoff arrays. (a) Spot printed on amine-functionalised surface shows uneven coffee ring pattern. (b, c) Liftoff polymer arrays with 20- and 10-μm openings. The relative thickness of the coffee ring inside the opening increases as the diameter decreases, yielding the most uniform deposition at 10-μm. Scale is the same for both micropatterned arrays. (d) Comparison of the percentage standard deviation of the mean fluorescence intensity for control *vs* 10-μm opening liftoff arrays, (e)–(g) Pseudo-colour of intensity of arrays in (a-c) for uniformity comparison. (h) Standard deviation associated with measurements of pixel intensities across arrays for successive dilutions. Inset: standard deviation for individual dilutions represented as a percentage of the mean intensity. Adapted with permission from reference [56]. Copyright 2007 American Chemical Society.

Hybrid surfaces with tailored hydrophobicity and hydrophilicity microfabricated using parylene “peel-off” stencils (polymer liftoff) have been demonstrated to improve DNA microarray reproducibility. These hybrid microarray surfaces were created by etching 10 μm or 20 μm diameter openings in a hydrophobic parylene-C film and then functionalising the area inside the openings with a hydrophilic aminopropyltriethoxysilane. These polymer liftoff arrays reduced the visible coffee-ring effect that is observed when DNA solutions dry on a hydrophilic aminosilane glass slide, which in turn reduced the standard deviations of microarray replicate experiments. The results showing the improvement in the reproducibility of DNA microarrays through the use of polymer liftoff surfaces are shown in Figure 7. The use of such novel hybrid surfaces could potentially increase the confidence in the data generated from microarray experiments, and be further exploited in electrowetting [54,55] and printing industries whereby control of surface hydrophobicity is crucial.

### 3.3. Patterned Lipid Bilayers for Immune Cell Activation

**Figure 8:**
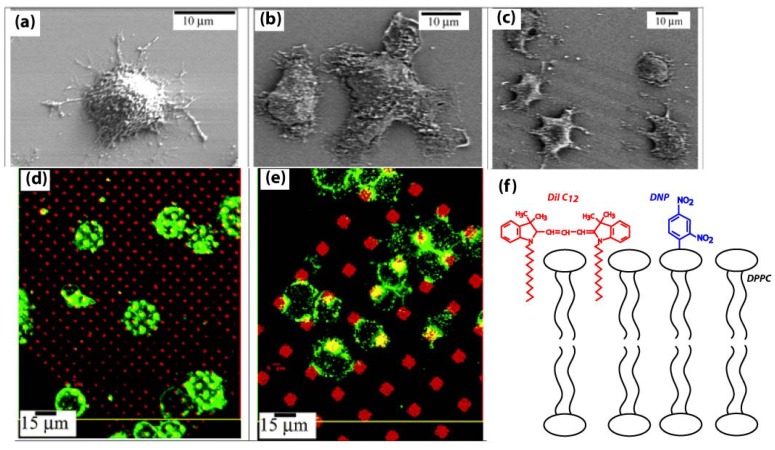
Scanning electron micrograph (SEM) images of RBL cells: (a) Resting RBL on a plain silicon surface with lamellipodia randomly spreading on the oxidised silicon substrate; (b) Stimulated RBL at the corner of four patterned squares of lipids; (c) Stimulated RBL over patterned lines of lipids. Gray lines indicate where the antigenic lipids were patterned. Interaction between RBL cells and the patterned lipid bilayer with (d) 1.5 μm pattern, 5 μm period, and (e) 6 μm pattern, 16 μm period. Confocal images of RBL cells sensitised with Alexa 488 anti-DNP IgE (green) on a 1,1'-didodecyl-3,3,3',3'-tetramethylindocarbocyanine perchlorate (DiIC_12_) fluorescently labeled lipid bilayer (red). (f) Schematic showing composition of lipid bilayer patterned in (d): 1,2-dipalmitoyl-sn-glycero-3-phosphocholine (DPPC), 1,2-dipalmitoyl-sn-glycero-3-phosphoethanolamine-N-[6-[(2,4-dinitrophenyl)amino]hexanoyl] (DNP-cap-DPPE) and DiIC_12_ with molar ratio 94.5:5:0.5. Dinitrophenyl (DNP, blue) is the antigen for stimulating the mast cells, while DiIC_12_ (red) fluorescently labels the lipid bilayer. Adapted with permission from reference [58]. Copyright 2003 American Chemical Society.

A key advantage of parylene “peel-off” micropatterning is the ability to pattern biomolecules in hydrated environments. This is critical for maintaining the bioactivity of proteins and especially bilayer structure of lipids. As the most stringent test of hydrated patterning, lipid bilayers have been successfully micropatterned using the parylene “peel-off” approach [57]. Orth and coworkers have created lipid bilayers patterns with different shapes and subcellular dimensions containing the antigen dinitrophenol (DNP) [58]. Immune cells (rat basophil leukaemia [RBL]) were activated by these antigen presenting lipids and extended lamellipodia in the direction along the lines and square patterns of lipids, shown in Figure 8a-c. Furthermore, the RBL cells displayed different morphologies when stimulated with lipids patterns of different shapes and sizes in Figure 8d-e. Lipid bilayers of heterogeneous compositions with different functionalities (e.g. presenting antigens, incorporating fluorescent dyes) could be patterned, as shown by the schematic in Figure 8f. The simplicity and adaptability of parylene “peel-off” for patterning lipids offer a convenient alternative to more complicated lipid patterning approaches such as photopolymerisation and electric field-induced reorganization, *etc.* [59,60], and could play a future role in lipidomics.

### 3.4. Patterning Biomolecules Inside Microfludic Channels

**Figure 9:**
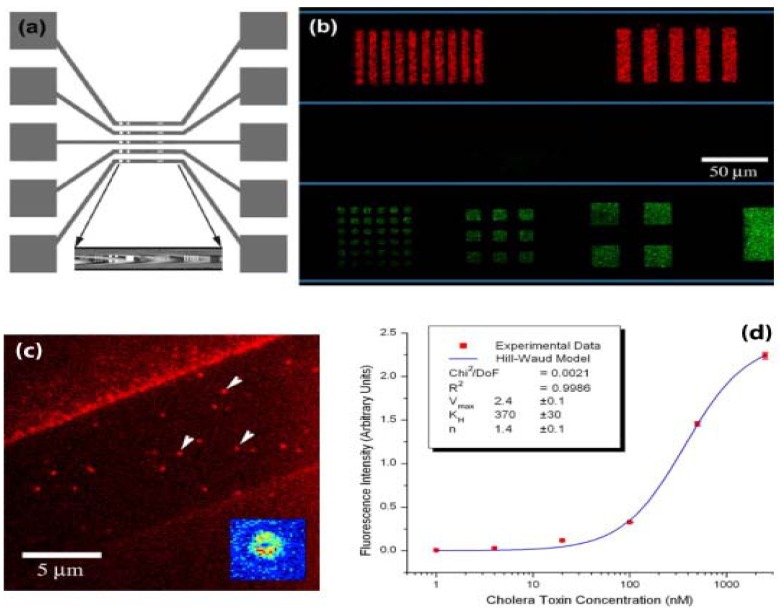
Supported lipid bilayer (SLB) arrays within microfluidic channels. (a) Top view scheme of designed microfluidic trenches and reservoirs and patterned features at the bottom of these trenches (coloured marks in central portion). (Inset) Bright field image of top view of patterned trench. (b) Alexa 594-cholera toxin subunit B (CTB) and Alexa 488-tetanus toxin fragment C conjugated toxin fragments segregated from a binary mixture and bound to SLB arrays in the microfluidic channels (the channel boundary is represented by the thin horizontal lines). (c) Isolated binding events for cholera toxin observed via total internal reflection fluorescence microscopy. Binding events (arrows) observed in patterned SLB (strip in center, 10 μm wide) without removal of parylene-C coating (brighter flanking regions). (Inset) False colour intensity plot of a single bound event. (d) Binding assays for CTB. Fluorescence intensity versus concentration plot for successive dilutions images of CTB incubated with patterned SLBs containing G_M1_ gangliosides. (Inset) Hill-Waud model fit parameters. Reprinted from reference [63]. Copyright 2005, with permission from Cell Press, Elsevier.

Microfluidic systems hold promise for portable bioassays and interrogation of biomolecules with minimal reagent and sample volume consumption [8,9,10]. The ability to pattern biomolecules inside the microfluidic channels allows for specific recognition of biological analytes. Patterning inside fluidic channels has been demonstrated by several groups utilising laminar flows or crossed flows to create combinations of biomolecules [6,61]. Parylene “peel-off” stencils can be addressed using fluidic channels for patterning multiple types of biomolecules inside microfluidics [62,63]. For example, Moran-Mirabal and coworkers have demonstrated patterning of gangliosides embedded in lipid bilayers inside microfluidic channels in Figure 9a. In Figure 9b-d, this biosensor device was used to detect cholera and tetanus toxin subunits, by measuring the signal from the fluorescently-labeled toxins that bound to the their natural target (gangliosides). Individual channel flows have been used to address openings in parylene stencil, so as the achieve selective spatial patterning of multiple biomolecules on a surface [62]. In the future, parylene “peel-off” could be combined with inkjet printing for higher multiplexing of biomolecular patterning inside microfluidic channels.

### 3.5. Cell Culture Arrays

**Figure 10:**
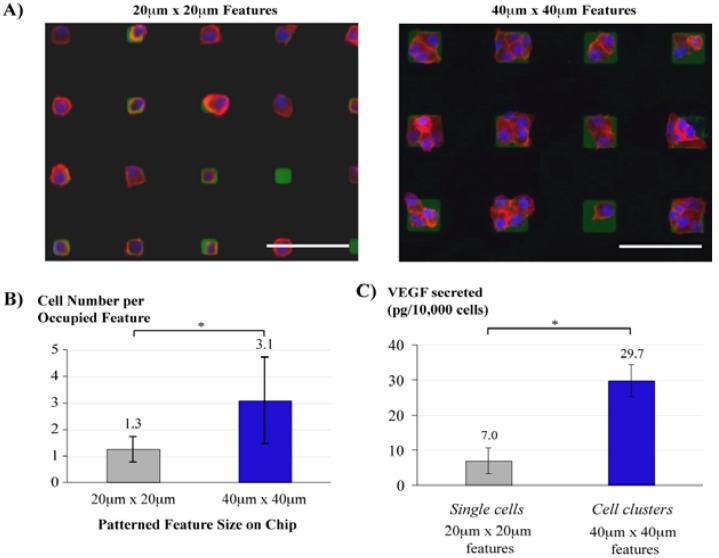
(a) Arrays of single cells and cell clusters as developed by culturing oral squamous carcinoma (OSCC3) cells on PeelArray chips with 20 μm × 20 μm (left) and 40 μm × 40 μm (right) features, respectively. Visualisation was performed by fluorescent staining of the tumour cells (nuclei [DAPI, blue], cytoskeleton [phalloidin, red]) and patterned fibronectin (anti-human fibronectin, green). (b) Average number of OSCC3 cells per fibronectin feature on the different chips as quantified by image analysis. (c) Normalised secretions of VEGF were significantly up-regulated in cell clusters compared to single cells devoid of cell-cell contact. (* p < 0.01). Reproduced and adapted from reference [53] (DOI: 10.1039/b908036h) by permission of The Royal Society of Chemistry.

Micropatterning is an attractive method to spatially control the cellular microenvironment and guide cell behaviour, useful for tissue engineering and cell studies. The cytocompatibility of parylene supports cell growth and cultures. The parylene “peel-off” approach has been demonstrated for directing neuronal cell growth [64], spatio-temporal control of cell-cell interactions for tumour angiogenesis studies [53], as well as co-cultures of different cell types [49]. In Figure 10, parylene “peel-off” stencils with different feature sizes (either 20 × 20 μm or 40 × 40 μm squares) enabled a facile method to control the level of cell-cell interaction by patterning single cell (average 1.3 cells per feature) or cell clusters (average 3.1 cells per feature). This is a simple and straightforward approach for patterning single cells compared to other more complicated techniques such as optical tweezers, dielectrophoresis or microfluidic flow capture. About 30,000 cells were uniformly patterned on a large area on each chip either as single cells or cell clusters, secreting enough proteins for analysis by enzyme-linked immunosorbant assay. The uniform patterning of the number of cell(s) per feature enabled the normalisation of vascular endothelial growth factor (VEGF) secretions by the cell numbers, and a correlation to be made between VEGF secretions and the level of cell-cell interactions as shown in Figure 10c.

**Figure 11:**
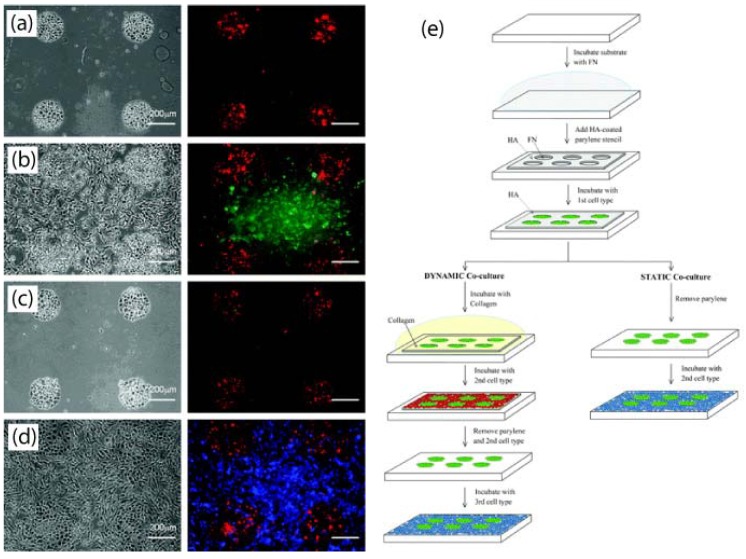
Light micrograph (left) and the corresponding fluorescent (right) images of the steps in the formation of dynamic co-cultures using parylene-C stencils. (a) A hyaluronic acid (HA) coated parylene-C stencil was reversibly sealed on fibronectin (FN) treated PDMS and seeded with mES cells. (b) The patterned co-cultures of mouse embryonic stem cells (mES) and AML12 hepatocytes. (c) To generate dynamic co-cultures, the stencil was gently peeled away, leaving the mES cells. (d) After depositing a layer of FN, a third cell type (NIH-3T3) was seeded on the exposed surface. (e) Schematic diagram of the process used to generate static and dynamic co-cultures. Reproduced and adapted from reference [49] (DOI: 10.1039/b706081e) by permission of The Royal Society of Chemistry.

Co-cultures of two or more cell types have been achieved with parylene stencils [49] as outlined in Figure 11. In the static mode, the first cell type is seeded onto the openings of the parylene stencil and afterwards the stencil is peeled off and the second cell type seeded onto the surrounding regions. In the dynamic mode, the parylene stencil is coated with collagen after the first cell seeding, and the second cell type is seeded onto the parylene stencil. Afterwards, the stencil can be peeled off to reveal the underlying region for seeding the third cell type. Such cell co-cultures can be important for mimicking the liver cellular microenvironment for drug testing, as well as culturing cells that respond to physical or chemical cues from its neighbouring cells.

### 3.6. Multilayer Parylene Stencils

**Figure 12:**
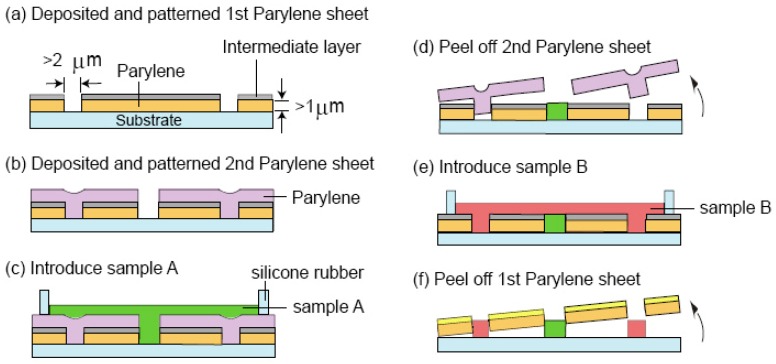
Process flow showing the selective patterning of two types of biomolecular samples using sequential multilayer parylene lift-off steps. Reprinted with permission from reference [50]. Copyright (2007) IEEE.

Surfactants (e.g., Tween-20) and protein (bovine serum albumin [BSA]) solutions can be applied as an intermediate separating layer to enable the creation of multilayer parylene films stack. Multilayer parylene stencils have extended the utility of the parylene “peel-off” micropatterning approach. For example, the total number of different cell types that could be sequentially patterned as co-cultures was increased from three to five using a three-layers parylene stencils compared to just a single layer stencil [51]. The schematic in Figure 12 illustrates an interesting work performed by Kuribayashi and coworkers, demonstrating patterning of two different biomolecules within close proximity of each other using a two-layers parylene stencil [50]. This can be an alternative patterning approach to laminar flow patterning using microfluidics for patterning two streams of biomolecules separated by subcellular distances [6]. The ability to position two different species with sub-micrometre interval could increase the packing density of biomolecules on microarrays, and improve throughput for screening more candidates per unit area. Furthermore, the work by Kuribayashi also creatively patterned a parylene sheet with the protein BSA using a top layer of parylene stencil. The stencil was peeled off and the bottom parylene sheet could be rolled up to form a microtube with patterned BSA on its surface. This proof-of-principle could possibly be modified in the future for engineering micropatterned artificial blood vessels.

### 3.7. Biophysical Studies and High-Resolution Optical Imaging

Parylene “peel-off” micropatterning is becoming increasingly popular for immobilising biomolecules with subcellular or sub-micrometre feature sizes onto surfaces for high resolution imaging in biophysical studies. Localised micropatterned features can spatially target or activate subcellular receptor-ligand interactions. When combined with high-resolution fluorescence microscopy techniques, biomolecules can be manipulated with nanoscale precision using these patterned surfaces, potentially elucidating the inner workings of cells and shedding new light on biophysical processes. In a study by Moran-Mirabal and coworkers, the binding and activity of cellulases have been quantified by fluorescence microscopy of Alexa Fluor 647 labeled cellulases moving along Alexa Fluor 488 labeled cellulose fibrils [65]. These fibrils were immobilised onto glass coverslips using parylene “peel-off” stencils. Likewise, microtubules have also been patterned onto surfaces for biophysical studies involving the translocation of kinesin along these tubules [66]. Through the use of parylene “peel-off” micropatterned DNP-lipid arrays, it was discovered that focal adhesion proteins connect the IgE receptors on the surface of immune cells to the cytoskeleton [67]. The spatially defined clustering of receptor-ligands corresponding to the periodicity of the pre-defined micropatterns can provide a convenient and quantitative evaluation of local signaling pathways.

## 4. Reactive Parylene Coatings

**Figure 13:**
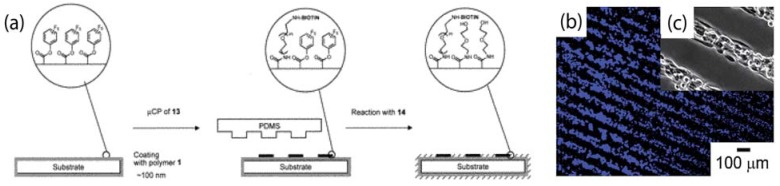
(a) Representation of the process that was used for mCP of the amino-functionalised biotin ligand 13 ((+)-biotinyl-3,6,9-trioxaundecanediamine) onto reactive parylene coatings with active ester group (shown as an example). A PDMS stamp, manufactured as a replica from a silicon master, was used for printing ligand 13 onto polymer 1 (parylene coating containing reactive ester group). The remaining surface area was then passivated by reaction with compound 14 (2-(aminoethoxy)ethanol). (b) Fluorescence micrograph of bovine aortic endothelial cells (BAEC) seeded for 24 h on a gold substrate (cell nuclei were stained with bis-benzimide). (c) Enlargement of optical micrograph of BAEC seeded for 24 h on a gold substrate. In all cases, the substrate was previously coated with polymer 1 and patterned with ligand 13 to control self-assembly of anti-integrin antibody. The width of each individual line is 50 μm. Adapted with permission from reference [32]. Copyright 2002 American Chemical Society.

Functionalised parylene have been deposited onto surfaces to confer active chemical functionalities. An application of such reactive parylene coatings is for patterning proteins and mammalian cells onto surfaces independent of the substrate material [32], so long as the material can be coated with parylene. In this work, Lahann and Langer have demonstrated patterning of integrin proteins and cells onto surfaces coated with functionalised parylene films containing either active ester groups or anhydride groups. Aminated-biotin was microcontact printed using PDMS stamps onto the parylene surface and allowed to react with the ester or anhydride groups, as illustrated in Figure 13. Afterwards, streptavidin-biotin conjugation chemistries were employed to immobilise integrins and cells onto the biotin micropatterns on parylene. Similar strategies for micropatterning surfaces coated with parylene containing reactive functional groups such as alkynes were also reported [33].

Reactive parylene coatings are also convenient for direct surface modification of biomedical implants. R-hirudin, a protein with anticoagulant properties have been covalently linked onto parylene coated surfaces of metallic implants, thereby increasing implant haemocompatibility [30,37]. Polymeric surfaces such as poly(vinylidenefluoride) can be coated with parylene with amine functional groups to covalently link fibronectin to the surface for improving osteoblast cells adhesion [68]. Temperature sensitive poly-N-isopropylacrylamide (pNIPAM) was grafted via atom transfer radical polymerisation onto reactive parylene surface, leading to temperature dependent tissue adhesion [29]. pNIPAM chain collapses at temperatures above the critical solution temperature (31 °C) to form a hydrophobic surface that promotes tissue adhesion. These studies indicate that reactive parylene coatings are versatile biomaterials for tailoring and engineering surface properties of biomedical implants.

### 4.1. Functionalised Microfluidic Channels

**Figure 14:**
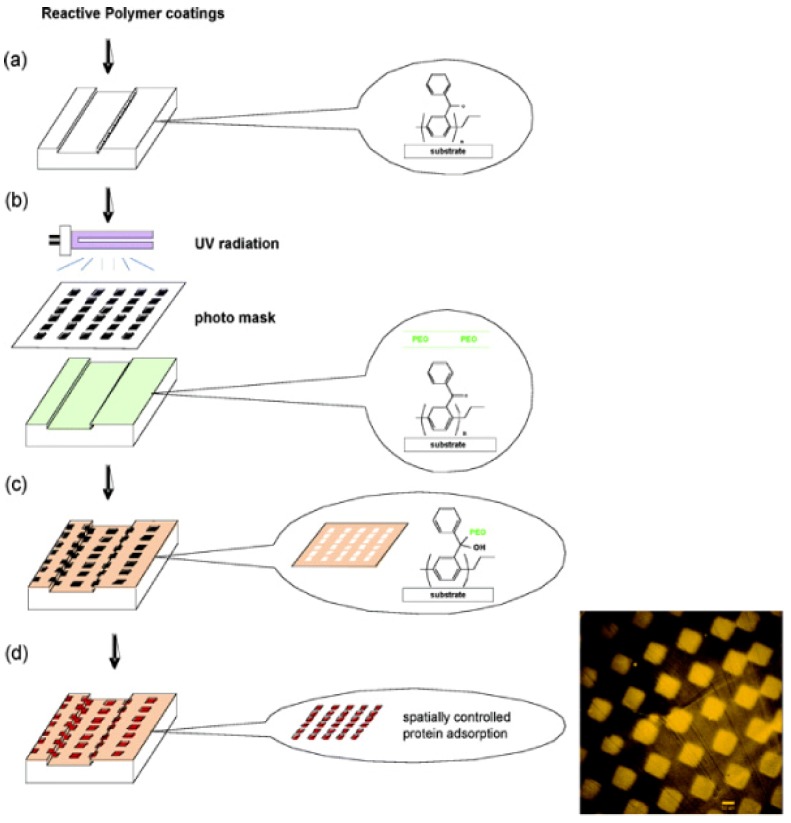
Spatially controlled protein adsorption via photopatterning of reactive coatings deposited within microchannels. (a) Reactive coatings are deposited via CVD polymerisation. (b) Photopatterning is conducted using a photomask. (c) After rinsing, PEO is selectively immobilised to areas that were exposed to UV radiation (areas surrounding squares). (d) The entire surface is incubated with protein solutions, but proteins preferentially adsorb to non-modified areas. Inset: Surfaces with square patterns of BSA adsorbed onto CVD/PEO-modified silicon substrate. Adapted with permission from reference [36]. Copyright 2005 American Chemical Society.

Phenylacetyl groups on parylene coatings can be photoactivated via ultraviolet light. The photoactivated group is then able to crosslink with polyethylene oxide (PEO) molecules in close proximity to the surface via C-H addition reaction to form PEO hydrogels [35]. The PEO polymerised regions resist protein adsorption and this method was demonstrated for selective three-dimensional micropatterning of proteins (BSA and fibrinogen) independent of topography inside parylene coated microfluidic channels [36], as shown in Figure 14.

Parylene films with amine groups have been used to functionalise the internal surface of microfluidics with CD31 antibodies for cell capture and sorting [69]. The CD31 antibodies interact with the antigens expressed on human umbilical-vein endothelial cells (HUVEC) and decrease the cells’ mobility. This cell sorter device was shown to successfully separate fractions of HUVEC from leukocytes that did not express the CD31 antigen, by exploiting differences in cell mobility inside the channels. Microfluidic devices capturing bovine aortic endothelial cells have also been demonstrated using anti-integrin antibodies functionalised inside the microfluidic channel via parylene reactive coatings [38].

### 4.2. Vapour Assisted Microstructures Using Replicas and Templates

**Figure 15:**
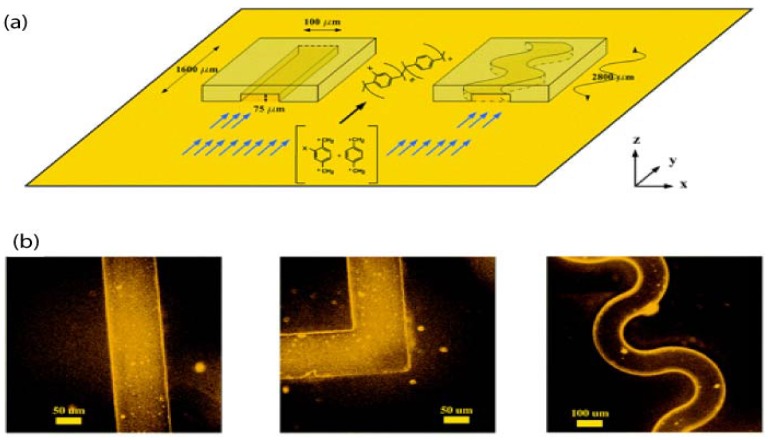
(a) CVD polymerisation within confined geometries of straight and meandering channels. (b) Fluorescence micrographs of sealed devices coated with parylene containing functional ketone groups, after immobilisation of hydrazide−biotin and self-assembly of fluorescent tetramethyl rhodamine isothiocyanate-conjugated streptavidin. Examples are shown in different channel geometries of 75 μm depth and 100 μm width. Adapted with permission from reference [71]. Copyright 2006 American Chemical Society.

By exploiting the conformal nature of parylene coatings, pre-defined channels and microgeometries in materials such as PDMS, have been used as replica and mask templates to assist the vapour deposition of parylene [70,71]. Figure 15a is a schematic illustrating the vapour-assisted deposition of parylene by using confined geometries in PDMS. Figure 15b shows the various microfluidic geometries and channel widths that can be created and the fluorescence indicates successful functionalisation of biotin and streptavidin on the internal fluidic surfaces. Openings with feature sizes as small as 25 μm wide separated by a 25 μm pitch have been patterned in parylene films using a replica [70]. X-ray photoelectron spectroscopy confirmed the presence of parylene in the centre of the microstructure, indicating the monomer vapour was able to reach deep within the replica structure during the CVD process [70]. These methods are simple and adaptable, solventless and lithography-free alternatives towards topologically and chemically designable microstructures from parylene. The advantage is that microfluidics fabricated this way can contain reactive functional groups inside the channels, which are suitable for surface modification with biomolecules later.

**Figure 16:**
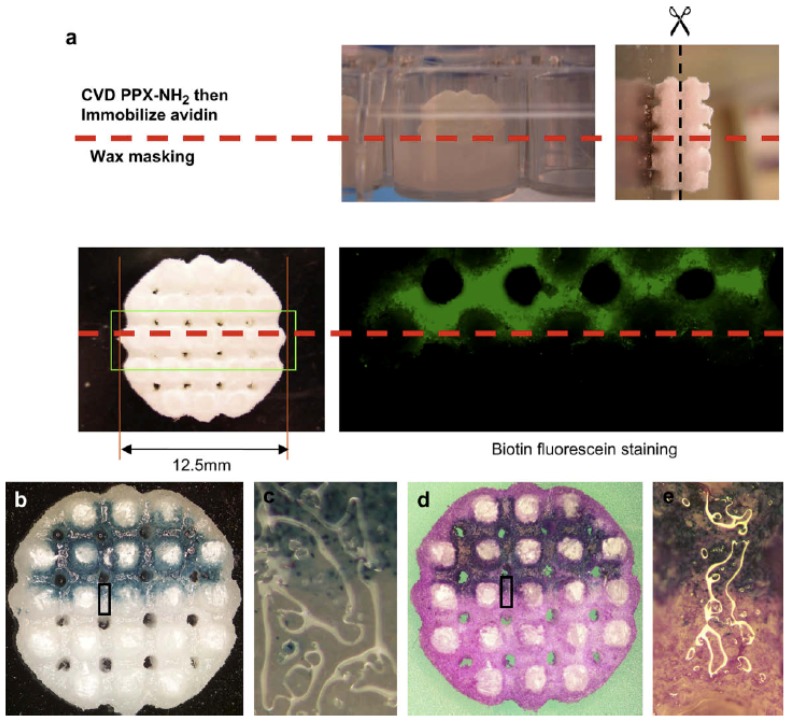
Spatial control of adenovirus immobilization in 3-D scaffolds. Reprinted from reference [72]. Copyright (2009), with permission from Elsevier.

(a) Half of each scaffold was embedded in low-melting wax to restrict the CVD treatment to only the non-masked regions for avidin immobilization. After wax removal, scaffolds were cut halfway down their heights and stained with biotin–fluorescein. The bound avidin was distributed on non-masked regions inside the scaffolds. (b) Biotinylated AdLacZ was immobilised in 3-D scaffolds before culturing with fibroblasts. After a 2 day culture period, scaffolds were cut halfway down their height and stained with X-gal. Transduced cells were distributed in the same regions as the bound avidin. (d) Counter staining with crystal violet illustrated that fibroblasts grew to confluence in the scaffolds. Transduced and non-transduced cells were controlled to distribute in two different regions with distinct interfaces. (c) and (e) are high magnitude images of interface regions in (b) and (d), respectively.

### 4.3. Three-Dimensional Scaffolds for Adenovirus Gene Delivery

Biomaterial (usually a biocompatible polymer) scaffolds hold potential for *in situ* gene delivery and subsequently provide a mechanical support for surrounding cells to grow during tissue reconstruction. However, most biomaterials lack active functional groups on their surface for the bioconjugation of biomolecules or viral vectors carrying the gene payload. To overcome this issue, thin films of reactive parylene coatings that are non-cytotoxic, biocompatible and mechanically robust can be deposited directly onto the biomaterial scaffolds to impart chemical functionalities to the surface. One such interesting work was performed by Wu and coworkers, shown in Figure 16a, whereby three-dimensional poly(e-caprolactone) scaffolds were selectively coated with parylene with amine functional groups to facilitate bioconjugation of adenoviruses to the scaffold [72]. The bottom half of the scaffold construct was protected by a low-melting wax from parylene deposition, and the wax was later melted away after parylene CVD. The viruses (delivery vectors for the beta-galactosidase gene) were localised to the top half of the scaffold and successfully transduced fibroblast cells (cultured on the scaffold) to express the galactosidase enzyme, as shown in Figure 16. This work demonstrates the feasibility of using reactive parylene films for localised bioconjugation to engineer biomaterial scaffold surfaces for tissue regeneration and gene delivery.

**Figure 17:**
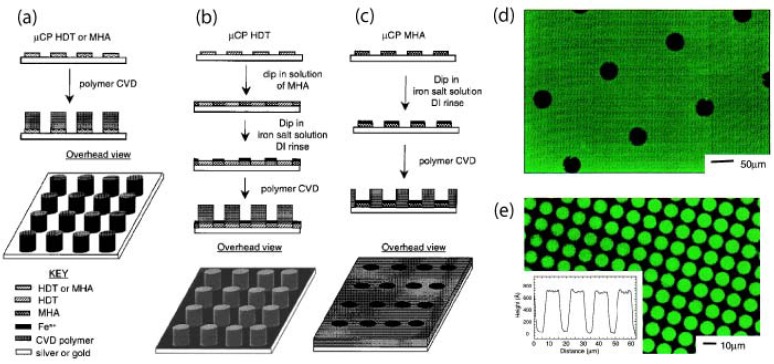
Use of mCP to control the growth of CVD parylenes and poly(p-phenylene vinylene) (PPV, a type of parylene) through (a) activation of the surface to polymer growth by mCP of hexadecanethiol (HDT) or 16-mercaptohexadecanoic acid (MHA), (b) deactivation of the printed regions of the surface to polymer growth by mCP of MHA followed by exposure to an iron salt solution and rinse step, and (c) deactivation of the unprinted regions of the surface to polymer growth by mCP HDT on the surface, depositing MHA on the unprinted regions, and exposing the entire surface to a solution of iron salt, followed by a rinse step. Photoluminescence from selectively grown CVD PPV films generated by combining mCP alkanethiols and a growth-inhibiting iron salt: (d) holes in the PPV film (35 μm in diameter) fabricated as shown in (b), using MHA in the printing step and iron(III) chloride as the growth-inhibiting salt; (e) an array of CVD PPV dots (12.5 μm in diameter separated by 2.5 μm) fabricated as shown in (c), using HDT in the printing step, MHA in the unprinted regions, and iron(II) sulfate as the growth-inhibiting salt. Adapted with permission from reference [19]. Copyright 2000 American Chemical Society.

## 5. Parylene Microstructures

### 5.1. Substrate-Selective Vapour Deposition

Substrate-selective deposition of parylene can be exploited for creating patterned parylene films without the use of lithography. Transition metals, such as iron, and their salts have been reported to inhibit parylene deposition by binding and deactivating the reactive monomer during CVD [20,73]. As shown in Figure 17, gold and silver surfaces that inhibit parylene deposition were microcontact printed with thiols to activate the surfaces for parylene deposition [19]. However, this surface activation can be reversed be passivating the surface with a layer of iron salts, thereby inhibiting parylene deposition again [19]. A systematic study of metals revealed interesting observations that certain metals such as titanium, copper, and nickel can selectively inhibit deposition some types of functionalised parylenes [73]. These studies could have future implications on surface modification and deposition of parylene onto metallic biomedical implants and prostheses.

### 5.2. Parylene Tubes and Microfluidics

**Figure 18:**
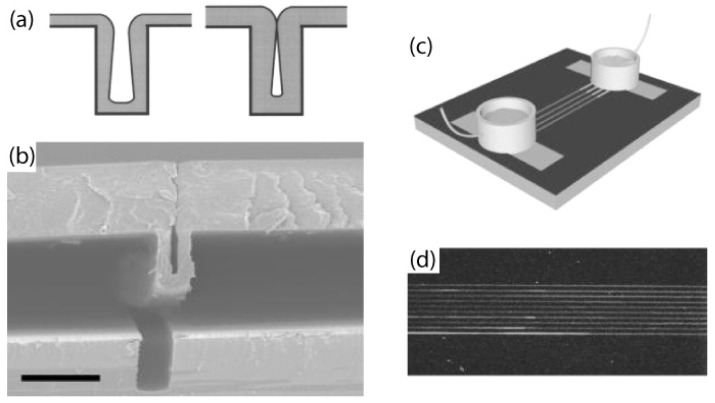
(a) Nonconformal step coverage of the high aspect ratio deep trench caused by lack of surface adatom migration. (b) Cross-sectional SEM of a partially delaminated fluidic channel from a high aspect ratio silicon template. Scale bar: 5μm (c) Schematic of the electrokinetic transport of rhodamine B. (d) Fluorescent micrograph of rhodamine B flow near the outlet of a channel. Reprinted with permission from reference [26]. Copyright [2002], American Institute of Physics.

Self-sealing parylene tubes can be created by depositing parylene onto high aspect ratio trenches that are etched into a silicon wafer [26,27]. At a critical thickness of parylene, the top of the trench pinches off to form a sealed parylene tube in Figure 18a. Centimetre-long tubes with inner cross-sectional widths of 400nm to 20μm have been created by this process, and tubes can be peeled off the silicon wafer shown in Figure 18b. The ends of the tubes can be connected to reservoirs using photocurable adhesive as seen in Figure 18c to create microfluidic channel. In Figure 18d, fluorescent rhodamine B dye was electrokinetically driven through the parylene tube channels without leakage by applying a voltage across the reservoirs. In addition to the obvious applications for microfluidics and biosensors, the biocompatibility and range of length-scale dimensions of these tubes suggests possible uses for biomedical implantable fluidic networks and artificial blood vessels.

**Figure 19:**
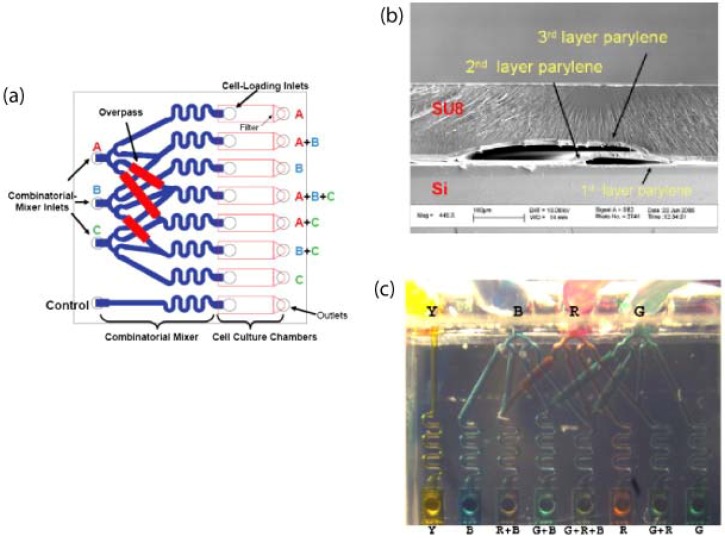
(a) Layout of the device design showing the components of the chip. The combinatorial mixer takes in three-input streams and delivers the seven different combinations into the cell culture-chambers. There are several microfluidic “overpass” structures that allow one microchannel to cross over other microchannels. One control channel that receives unmixed input stream is included. Cells can be grown inside the culture-chambers. The chip has a dimension of 1 cm × 1 cm. (b) SEM image of the cross-section of the microfluidic overpass. The overpass has two-level microfluidic channels and such structures will allow two fluidic streams to be separated spatially at the overpass. (c) Combinatorial mixer operated at 0.1μL/min flow rate. As flow rate goes down, molecules have more time to diffuse across the channel for better mixing. Reprinted from reference [74]. Copyright (2008), with permission from Elsevier.

**Figure 20:**
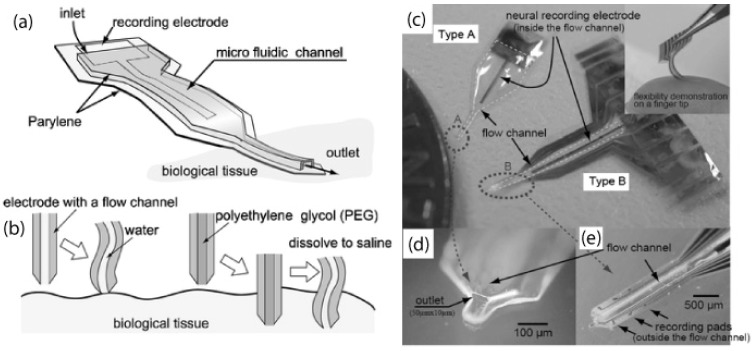
The concept of the neural probe. (a) The fluidic channel is used to improve the stiffness of the probe during insertion. The channel also allows drugs to be injected into the cells and the neural signals from them to be measured. (b) Compared with a channel filled with water, the channel filled with PEG allows smoother insertion; PEG dissolves after the implantation and the probe's flexibility is recovered in the tissue. Photos of the probes. (c) Type A is a single channel electrode. Type B has 6+1 recording channels (six channels are outside and one is inside the fluidic channel) and work for extracellular neural recording. (d) and (e) are close-up views of the tips of type A and B. Reproduced from reference [75] (DOI: 10.1039/b417497f) by permission of The Royal Society of Chemistry.

More complicated architectures of three-dimensional microfluidic channels have been realised by monolithic fabrication with a photoresist sacrificial layer. These devices, shown in Figure 19, are used for combinatorial mixing of flows and cell culture [74]. Parylene microfluidic channels have also been micromachined into neural probes with electrodes for recording neural signals [75], such in Figure 20. These fluidic channels allow for the injection of chemicals into the brain tissue, and can be filled with a water-soluble polymer such as polyethylene glycol (PEG) to improve mechanical stability of the otherwise flexible neural probe.

### 5.3. Micromachined Structures and Membranes

**Figure 21:**
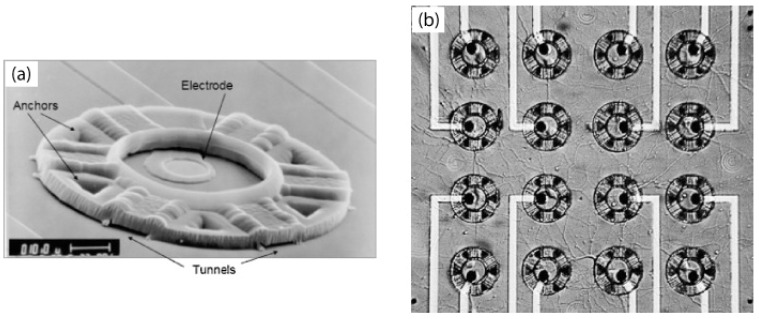
(a) SEM of the final neurocage design. The major parts of the neurocage are labeled. A neuron is placed in the central chimney region, near the electrode. Axons and dendrites are free to grow though the tunnels to synapse with other neurons. The cage is made out of 4 μm parylene, a biocompatible polymer. Low-stress silicon nitride insulates the gold electrode and leads. Scale bar: 10 μm. (b) 10 days old neuronal culture on chip. Neuron bodies (soma) are trapped in cages, with fine axonal processes outgrowth through the tunnels forming a rich network with surrounding neurons. Reprinted from reference [76]. Copyright (2008), with permission from Elsevier.

Neurocages have also been micromachined entirely out of parylene for supporting neuronal cultures and integrating the neurons with electrodes for electrical stimulation and recording action potentials [76,77,78], show in Figure 21. The large central chimney region would isolate the neuronal body (soma), while permitting fine axonal extensions out of the tunnels to form networks with neighbouring neurons in Figure 21a. Arrays of neurocages interfaced with electrodes have been utilised for mapping functional neural networks in Figure 21b.

**Figure 22:**
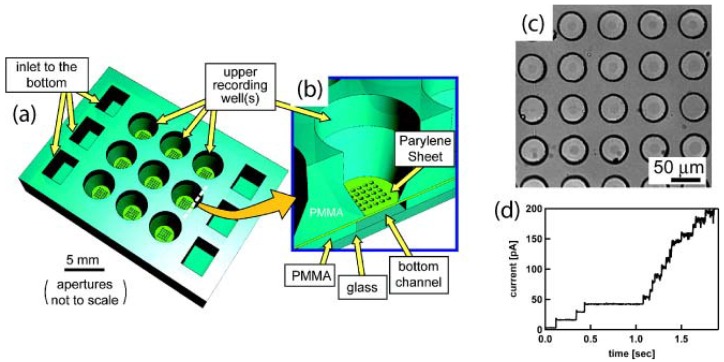
Schematic of the lipid bilayer array chip. Adapted with permission from reference [79]. Copyright 2008 American Chemical Society.

Lipid bilayers can be formed across apertures (~40μm) in microfabricated parylene membranes [79,80]. These parylene membranes are incorporated between two polymethylmethacrylate (PMMA) plates to form recording wells as shown in Figure 22a-c. The lipid bilayers in the recording wells serve as biomimetic cell membranes whereby channel proteins such as α -hemolysin and gramicidin, can be inserted into the bilayer. Ion exchange through the channel proteins can measured as currents, demonstrated by the result in Figure 22d. These biomimetic membranes can be used to investigations of ligand and voltage gated ion channels and testing their responses to drugs.

(a) Overview and (b) close-up of sectional view at the recording well. A thin sheet of parylene with microfabricated apertures was sandwiched between two PMMA plates that serve as upper recording wells and bottom channels. Planar lipid bilayers were formed across the apertures by first filling the upper wells with buffer, then introducing lipid solution and buffer into the bottom channel. (c) Microscopic image of 25 planar bilayers formed in an array of 47 μm diameter apertures. The circular zone inside each aperture delimits the lipid bilayer. (d) Current through α-hemolysin membrane pore protein reconstituted in a single well device with 100 apertures having 40 μm diameter. Stepwise increase of channel current was observed at 60 mV voltage clamp condition, indicating the insertion of channel proteins.

## 6. Future Outlook and Conclusions

Surface chemical modification can be used for precisely controlling spatial location of biomolecules and cell behaviour. For this purpose, parylene is a versatile biomaterial with unique properties such as biocompatibility, pinhole-free conformal coating, low cytotoxicity, chemical inertness and resistance to swelling in aqueous environments, which are beneficial for biological applications. Micropatterning with parylene “peel-off” stencils offers the advantages of patterning biomolecules in hydrated environments, with high uniformity over a large area, and a minimal sub-100nm nanoscale resolution. This approach is particularly useful for patterning chemically and biologically sensitive molecules and helping to preserve their conformation and bioactivity. Multi-component arrays of biomolecules and their combinations can enable the study and mapping of the myriad of receptor-ligand interactions that occur in nature. The use of reactive parylene coatings with functional groups creates possibilities for surface modification of virtually any material, to create bioactive and biocompatible surfaces on devices and structures. Parylene as a biomaterial offer numerous possibilities in the fields of tissue engineering, biomedical implants, biosensors and biophysical studies. For example, reactive parylene coatings and parylene “peel-off” micropatterning may be combined for surface engineering in the future to mimic and re-create cellular microenvironments such as artificial blood vessels and blood-brain barriers, as well as improve the interfacing of biomedical implants with the host body. Micropatterned combinatorial arrays of proteins and lipids may open up new possibilities for studies in proteomics and lipidomics. Spatially patterned biomolecular arrays can be combined with high resolution imaging microscopy to elucidate subcellular biophysical processes. The versatility of parylene has made it an interesting biomaterial for use in controlling the biochemistry and topography of devices and systems for increasing numbers of biotechnology and biological research applications.
